# *“Everything the hujur tells is very educative but if I cannot apply those in my own life then there is no meaning”*: a mixed-methods process evaluation of a smoke-free homes intervention in Bangladesh

**DOI:** 10.1186/s12889-022-14283-6

**Published:** 2022-10-11

**Authors:** Cath Jackson, Zunayed Al Azdi, Ian Kellar, Noreen Dadirai Mdege, Caroline Fairhurst, Tarana Ferdous, Catherine Hewitt, Rumana Huque, Anna-Marie Marshall, Sean Semple, Aziz Sheikh, Kamran Siddiqi, MCLASS II trial team, Steve Parrott, Abdullah Sonnet, Shilpi Swami, Han-I. Wang, Qi Wu

**Affiliations:** 1 Department of Health Sciences, University of York, York, UK; 2 Valid Research Ltd, Wetherby, UK; 3 ARK Foundation, Dhaka, Bangladesh; 4 School of Psychology, University of Leeds, Leeds, UK; 5 Institute for Social Marketing and Health, University of Stirling, Stirling, Scotland, UK; 6 Usher Institute, University of Edinburgh, Edinburgh, Scotland, UK

**Keywords:** Tobacco, Second-hand smoke, Smoke free homes, Faith, Mosque, Intervention, Process evaluation, Bangladesh

## Abstract

**Background:**

Second-hand smoke exposure from tobacco significantly contributes to morbidity and mortality worldwide. A cluster RCT in Bangladesh compared a community-based smoke-free home (SFH) intervention delivered in mosques, with or without indoor air quality (IAQ) feedback to households to no intervention. Neither was effective nor cost-effective compared to no intervention using an objective measure of second-hand smoke. This paper presents the process evaluation embedded within the trial and seeks to understand this.

**Methods:**

A mixed method process evaluation comprising interviews with 30 household leads and six imams (prayer leader in mosque), brief questionnaire completed by 900 household leads (75% response), fidelity assessment of intervention delivery in six (20%) mosques and research team records. Data were triangulated using meta-themes informed by three process evaluation functions: implementation, mechanisms of impact and context.

**Results:**

IMPLEMENTATION: Frequency of SFH intervention delivery was judged moderate to good. However there were mixed levels of intervention fidelity and poor reach. Linked Ayahs (verses of the Qur’an) with health messages targeting SHS attitudes were most often fully implemented and had greatest reach (along with those targeting social norms). Frequency and reach of the IAQ feedback were good. MECHANISMS OF IMPACT: Both interventions had good acceptability. However, views on usefulness of the interventions in creating a SFH were mixed. Individual drivers to behaviour change were new SFH knowledge with corresponding positive attitudes, social norms and intentions. Individual barriers were a lack of self-efficacy and plans. CONTEXT: Social context drivers to SFH intervention implementation in mosques were in place and important. No context barriers to implementation were reported. Social context drivers to SHS behaviour change were children’s requests. Barriers were women’s reluctance to ask men to smoke outside alongside general reluctance to request this of visitors. (Not) having somewhere to smoke outside was a physical context (barrier) and driver.

**Conclusions:**

Despite detailed development and adaption work with relevant stakeholders, the SFH intervention and IAQ feedback became educational interventions that were motivational but insufficient to overcome significant context barriers to reduce objectively measured SHS exposure in the home. Future interventions could usefully incorporate practical support for SFH behaviour change. Moreover, embedding these into community wide strategies that include practical cessation support and enforcement of SFH legislation is needed.

**Trial registration:**

Current Controlled Trials ISRCTN49975452

**Supplementary Information:**

The online version contains supplementary material available at 10.1186/s12889-022-14283-6.

## Background

Exposure to second-hand tobacco smoke (SHS) is estimated to cause 1.2 million deaths and loss of 11 million disability-adjusted life years worldwide every year [1]. Our focus was Bangladesh and SHS exposure in homes. In a recent study of 1746 households in Mirpur, Dhaka, over half (55%) self-reported that smoking by household members and visitors was permitted inside the home [2]. Unfortunately, evidence of effective interventions in South Asia to reduce SHS exposure in the home is lacking [3–5]. Moreover, poor reporting means that the intervention elements with greatest efficacy are difficult to identify [3–5].

International literature shows an association between religious faith and reducing or eliminating smoking behaviours [6–12] with proposed mechanisms including the idea of leading a “puritanical” life, having spiritual strength to resist temptations for future benefit, and being part of a social network of people who lead healthy lives. Relatedly, religious leaders are often highly respected and trusted by their communities [7–12]. Together, these suggest that religious teachings, settings and leaders offer potential to deliver tobacco control interventions.

In Bangladesh, 89% of the population is Muslim [13]. Islamic teachings focus on principles of minimising harm to individuals and society; and maximising opportunities for individual and collective well-being [9]. As such, smoking is discouraged, although whether it is decreed as *mukrooh* (discouraged) or *haram* (prohibited) varies [9]. To date, very few evaluations of Islamic faith-based interventions targeting smoking behaviours have been undertaken [11, 14, 15].

A 2018 Cochrane review of interventions to promote smoke-free homes (SFH) reported that 24 of 78 included studies found statistically significant reductions in children’s SHS exposure [3]. No one intervention strategy was identified as the gold standard. Successful strategies included motivational interviewing, brief counselling, nicotine replacement therapy for smoking cessation for parents who smoke, and feedback on markers of SHS exposure including the use of indoor air quality (IAQ) feedback. IAQ feedback offers participants objectively measured information on the impact that smoking has on concentrations of air pollutants in their homes to motivate them to reduce or stop smoking inside. This has been effective in reducing SHS in homes and/or children’s biomarkers of SHS exposure in several trials across settings and formats, including immediate and delayed feedback [16–22].

We conducted a three-arm cluster randomised controlled trial, MCLASS (Muslim Communities Learning About SHS) II, in 45 mosques from the Mirpur area of Dhaka, to evaluate effectiveness and cost-effectiveness of a community-based SFH intervention delivered in mosques with (*n* = 16) or without (*n* = 14) IAQ feedback in reducing exposure to SHS in the home [23, 24]. Both interventions are described in Table 1. Mosques in the control arm (*n* = 15) received no intervention. We found that at 3- and 12-months post randomisation there were no significant differences on mean 24-h household airborne fine particulate matter (< 2.5 microns in diameter [PM2.5]) concentration between the SFH intervention, with or without IAQ feedback, and no intervention. The interventions were also not cost-effective when compared to no intervention. We therefore concluded that these interventions could not be recommended for Bangladesh [24]. In this paper, we present the findings from our embedded process evaluation [25], to understand their lack of influence on trial outcomes.

**Table 1:** Description of the content and delivery of SFH and IAQ feedback interventions

**Fig. 1:**
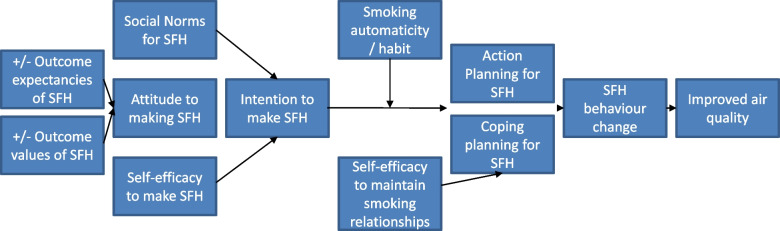
Intervention Programme Theory. *Note*. SFH is smoke-free home: smokers do not smoke inside, non-smokers request residents and visitors to smoke outside

**SFH intervention**
**CONTENT:** A set of 12 health messages relating to smoking and SHS exposure, each supported by at least one verse (Ayah) from the Qur’an, or an Islamic faith-based decree. The messages were developed through a set of iterative workshops involving Islamic scholars, public health professionals and behavioural scientists [26]. They addressed key barriers and drivers of smoking behaviours (attitudes, self-efficacy, social norms, intention formation, action and coping planning, see Fig. 1 and Additional file 1).
**DELIVERY:** Imams and khatibs were trained in a half-day session on the intervention and its delivery including detailed guidance on linking the messages and Ayahs. They then delivered the messages and Ayahs in the form of Khutbah (formal sermon preached by the imam in Arabic) to those attending Friday Jumu’ah prayer over 12 weeks (one linked Ayah-message per week). They also distributed copies of a short SFH booklet to their congregation in any way they saw best. The booklet contained a brief description of the 12 linked Ayahs-messages.
**IAQ feedback**
**CONTENT:** A two-page personalised leaflet designed in consultation with community members. It contained feedback on the air quality (PM_2·5_ concentration) measured within a home at baseline using the Dylos DC 1700 (Dylos, California, USA), an optical particle counter validated for use in domestic settings. Specifically feedback comprised a comparison of the 24-h mean PM_2·5_ concentration measured in the home to the World Health Organization (WHO) guidance limit of 25 μg/m^3^ [27], the total time the IAQ was above this guidance limit, and the maximum concentration measured during the 24-h measurement period. It included graphical information on how smoking activity impacted on IAQ over the 24-h measurement period (with classifications: hazardous if > 150 μg/m^3^, unhealthy if 36–150 μg/m^3^, moderate if 12–35 μg/m^3^, and good if < 12 μg/m^3^), information about the adverse effects of SHS exposure, recommendations to reduce SHS exposure in the home, and a target that was achievable by implementing SFH rules within the home.
**DELIVERY:** Trial field investigators delivered and discussed the personalised IAQ feedback with members of the households in person (in their homes) in approximately 10 min. Both the SFH manual and IAQ feedback leaflet are available here.

## Methods

### Overview of study design

This was a mixed method process evaluation conducted November 2018 to January 2019. It comprised interviews with household leads (trial participants) and imams (prayer leader in mosque), a brief questionnaire administered to household leads, fidelity assessment of intervention delivery and research team records. Findings from the different data sets were triangulated using meta-themes [28] based on the UK Medical Research Council’s [25] three process evaluation functions:

- Implementation – what is delivered (frequency, fidelity, reach)?
- Mechanisms of impact – how does the delivered intervention produce change? (intervention acceptability and usefulness, individual barriers and drivers to SHS behaviour change)
- Context – how does context affect implementation and outcomes? (social and physical context barriers and drivers to intervention implementation, and to SHS behaviour change)

SHS behaviour change included smokers not smoking inside the home and non-smokers requesting residents and visitors to smoke outside.

### Interviews

#### Participants

Semi-structured interviews were conducted post-intervention (at 3-month follow-up) with a sample of 30 household leads (14 in SFH arm, 16 in SFH + IAQ arm). Household leads were the nominated trial participant for participating households (*n* = 1801: 560 SFH, 640 SFH + IAQ, 601 control) where at least one adult resident was smoking regularly, at least one adult resident was a non-smoker and at least one resident attended a participating mosque. They were recruited to the trial at the mosque or through a home visit. We purposively selected household leads for interview to include men and women, smokers and non-smokers, with different descriptions of smoking in the home at 3-month follow-up (see Table 2). All imams who delivered the SFH intervention in six randomly selected mosques (3 from each intervention arm) were interviewed once intervention delivery was complete.

**Table 2:** Demographic characteristics and smoking/SFH status of interview participants

Characteristic		SFH (*n* = 14)		SFH + IAQ (*n* = 16)		All (*n* = 30)	
		**Men** **(** ***n*** ** = 10)**	**Women** **(** ***n*** ** = 4)**	**Men** **(** ***n*** ** = 10)**	**Women** **(** ***n*** ** = 6)**	**Men** **(** ***n*** ** = 20)**	**Women** **(** ***n*** ** = 10)**
Age, years	18–25	2	0	0	0	2	0
	26–35	5	1	5	0	10	1
	36–45	2	2	1	3	3	5
	> 45	1	1	4	3	5	4
Education, total years	No education (0)	1	2	3	2	4	4
	Primary (1–5)	4	1	4	3	8	4
	Secondary (6–10)	2	1	3	1	5	2
	Higher secondary (10–12)	2	0	0	0	2	0
	University (> 12)	1	0	0	0	1	0
Self-reported smoking status (at baseline)	Smoker	10	0	10	0	20	0
	Non-smoker	0	4	0	6	0	10
Description of smoking in the home (3-month follow-up)^a,b^	Nobody smoking	7	3	6	3	13	6
	Still some smoking	3	1	3	2	6	3
	Lots of smoking	0	0	1	1	1	1

^a^All described smoking in the home at baseline

^b^These descriptions may differ from the objective air quality data collected in the trial

Two-thirds of household leads were men (*n* = 20), and a similar proportion was aged < 45 years (*n* = 21). Over two-thirds (*n* = 24) had no/only primary (1–5 years) education. At baseline, all men self-reported as smokers; no women were smokers. About two-thirds of participants (*n* = 19) described their homes as smoke-free by 3-month follow-up, defined as not permitting residents or visitors to smoke inside the home. The rest (*n* = 11) described some/lots of smoking still occurring at home.

All six imams were non-smokers (a pre-requisite of their mosque’s inclusion in the trial). They had been an imam for between 6 and 35 years, and 2 to 22 years in their current mosque. The size of their congregation during Jum’ah prayers (a spiritually significant prayer offered during midday on Friday attended by men) varied from 800 to 4500 men.

#### Data collection

Interviews were conducted in Bengali face-to-face in the household lead’s home or at the imam’s mosque. All participants provided written informed consent before the interview commenced. Interviews with household leads explored interaction with the SFH intervention/IAQ feedback, views about the intervention(s), impact on SHS behaviours as well as individual or context barriers and drivers to creating a SFH (Fig. 1). These lasted 8–27 min. Interviews with imams explored acceptability of the SFH intervention, and experiences of delivery including individual or context barriers and drivers. These lasted 25–53 min. All interviews were digitally audio-recorded.

#### Data analysis

Interviews were transcribed verbatim, translated into English and checked by the interviewers. The data were subjected to Framework analysis [29] by two researchers (ZAA, CJ). Excel 365 facilitated data management.

An English language thematic framework was developed for each dataset based on the three process evaluation functions (implementation, mechanisms of impact, context) and their components (e.g. acceptability, social context barriers to SHS behaviour change). A sample of randomly selected interview transcripts (seven–household lead, two–imam) were used to further refine the framework, e.g. identify examples of social context barriers. The frameworks were piloted with more transcripts (three-household lead, one-imam) before finalising. The data were then charted into the relevant frameworks. Summaries of participant responses and verbatim quotes were entered. Both sets of charted data were then reviewed and interrogated to compare views, seek patterns, connections, and explanations within the data. Descriptive findings documents were written, organised by the components of the three process evaluation functions.

### Questionnaire

#### Participants and data collection

Household leads in the two intervention arms (SFH: 387 men, 33 women; SFH + IAQ: 461 men, 19 women; 75% response both arms) completed a short process evaluation questionnaire, administered face-to-face by a researcher at 3-month follow up). It asked questions on which components of the SFH intervention/IAQ feedback participants had received and perceived intervention usefulness.

#### Data analysis

Yes/no/don’t know responses were used for the intervention receipt questions. Perceived intervention usefulness was scored on a 7-point Likert scale from 1 (not at all useful) to 7 (extremely useful). Scores of 5 and above were classified as useful. Data were analysed using frequencies and proportions.

### Fidelity assessment

#### Data collection

Delivery of the SFH intervention was observed in six (20%) randomly selected mosques. Trained researchers conducted these checks and completed a fidelity index. Imams had previously received training on delivering the linked Ayahs (verses from the Qur’an) and health messages. They were unaware that they were being observed. In three mosques, delivery of Ayahs-messages scheduled for odd numbered weeks (1,3,5 etc.) were checked. In the other three mosques, Ayahs-messages scheduled for even numbered weeks (2,4,6 etc.) were checked. Each item in the index corresponded with the 12 weeks of Ayahs-messages targeting five key barriers/drivers to SHS behaviours (see Fig. 1 and Additional file 1). Delivery of each Ayah-message was scored 0–not implemented, 1–Ayah recited with no message, 2–Ayah recited with partial explanation of message, 3–Ayah recited with more than partial explanation but not full explanation of message, and 4–fully implemented. Definitions were provided for each Ayah-message (available from authors on request).

#### Data analysis

For each mosque, a total fidelity score was computed by summing the scores for Ayahs-messages from 0 (did not implement any Ayahs-messages) to 24 (all assessed Ayahs-messages were fully implemented). For each target barrier/driver (Fig. 1), we counted the number of times the Ayah-message was fully/partially/not implemented and divided this by the total number of opportunities for full implementation, for example, for “attitude” total number is 12 (3 mosques × 4 Ayahs-messages).

### Research team records

#### Data collection

Records were collected from mosques on their self-reported delivery of the SFH intervention. Field investigators self-recorded delivery of the IAQ feedback and a signature from the recipient was collected.

#### Data analysis

Counts and percentages were calculated for both delivery items.

### Triangulating findings

To triangulate the findings from the different datasets, the key findings for each intervention (SFI, IAQ feedback) from each dataset were displayed in a triangulation matrix (Additional files 2, 3) organised by the three meta-themes [28]: implementation, mechanisms of impact and context [25]. For each meta-theme, one or more datasets provided findings. Where there was more than one, these were compared to consider if they were convergent (in agreement), complementary (partial agreement), contradictory (disagreement) or silent (findings do not occur in a dataset but may have been expected to do so) [28].

## Findings

### Implementation

#### Frequency of SFH intervention delivery was judged moderate to good. There were mixed levels of intervention fidelity and poor reach. Ayahs-messages targeting attitudes were most often fully implemented and had greatest reach (along with those targeting social norms)

Records showed that 29 of the 30 mosques (97%) reported delivering all 12 weeks of the SFH intervention. The other mosque delivered 10 weeks. Imams typically reported they had delivered “almost all” of the SFH intervention as instructed, during Jum’ah prayer, before Khutbah (formal sermon preached by the imam in Arabic before the prayer) usually for 5–10 min. Two admitted to not delivering all 12 weeks. All described using other opportunities to share the Ayahs-messages in the mosque including in the Madrasas (educational institutions teaching Islamic subjects) and Maghrib (evening) prayers.

Whilst these convergent record and interview data indicated moderate-to-good frequency of intervention delivery, the questionnaire data revealed poor intervention reach. Only half of men in both intervention arms reported receiving the SFH intervention (SFH 49.4%; SFH + IAQ 55.5%). Women typically do not attend Friday prayers, so were asked if any family members had heard the Ayahs-messages. Once again, only half reported yes (SFH 51.5%; SFH + IAQ 52.6%). The interview data were more positive. All but three men reported having received the SFH intervention and only one woman was unaware of family members receiving it. For those men whom the intervention did reach, this was during Friday Jum’ah prayers (SFH 99.5%; SFH + IAQ 99.6%), with all women mentioning this for family members. Less than 3% of men reported receiving the SFH intervention in other mosque sessions. This reach via Friday prayers was confirmed in the interview data, thus both data sets supported the imams’ delivery accounts.

*The imam said directly, “Never smoke at home.” When he was delivering Khutbah, that time he talked about it.*

[Man, SFH intervention, nobody smokes in home at 3-month follow-up]

*Yes. I have come to know about it from my younger son. He goes to Jumu’ah always. I need not send him, he goes for his prayers by himself. Hujur* (prayer leader at the mosque) *tells many Hadith* (silent approvals of the prophet Muhammad) *and gives speeches on smoking.* [Woman, SFH intervention, nobody smokes in home at 3-month follow-up]

Regarding the detail of what was delivered by the imams, the mean fidelity score across six mosques was 19.6 (SD 2.51, range 16–22 of maximum 24). Ayahs-messages best delivered targeted attitudes and were 75.0% fully implemented. Ayahs-messages targeting self-efficacy and coping planning were 66.67% fully implemented. Ayahs-messages targeting social norms and intention formation-action planning were only 50.0% fully implemented (see Table 3).

**Table 3:** Fidelity to delivery of SFH intervention

	**Level of implementation (%)**					
**Target barrier/driver, n (%)**	**Full**	**Partial – level 3**	**Partial – level 2**	**Partial – level 1**	**Not implemented**	**No data** ^**a**^
Attitude, *n* = 12	9 (75.0)	1 (8.3)	0 (0.0)	1 (8.3)	1 (8.3)	0 (0.0)
Self-efficacy, *n* = 6	4 (66.7)	1 (16.7)	1 (16.7)	0 (0.0)	0 (0.0)	0 (0.0)
Coping planning, *n* = 6	4 (66.7)	1 (16.7)	1 (16.7)	0 (0.0)	0 (0.0)	0 (0.0)
Social norms, *n* = 6	3 (50.0)	0 (0.0)	1 (16.7)	0 (0.0)	1 (16.7)	1 (16.7)
Intention formation – action planning, *n* = 6	3 (50.0)	3 (50.0)	0 (0.0)	0 (0.0)	0 (0.0)	0 (0.0)

Delivery of each Ayah-message was scored 0–not implemented, 1–Ayah recited with no message, 2– Ayah recited with partial explanation of message, 3- Ayah recited with more than partial explanation but not full explanation of message, 4-fully implemented. Ayahs-messages linked to attitudes were scheduled for delivery in four weeks. The other four target barriers/drivers were scheduled for two weeks each

^a^No assessment as this was scheduled during the Eid festival

Interview and questionnaire data partially confirmed this. Imams described focusing particularly on the Ayahs-messages about risks of SHS to children, pregnant women, and others (targeting attitudes and social norms). This preference was unrelated to the ease/difficulty of delivery (they were confident with all 12). Instead, they believed their congregation were interested in learning about this, given that it is not usually spoken about in the mosques.

**Fig. 2:**
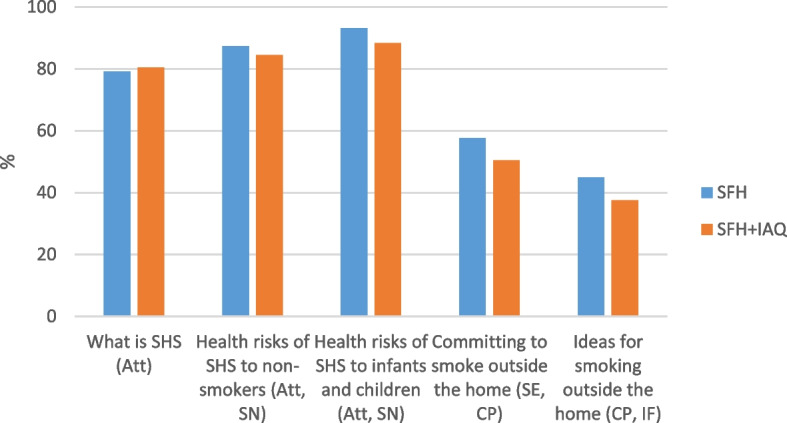
Percentage recall (reach) of SFH intervention Ayahs-messages by men who had received the SFH intervention. *Note*. Att = attitude, SN = social norm, SE = self-efficacy, CP = coping planning, IF = intention formation

These were also the Ayahs-messages that men most recalled hearing (79.1% to 94.8%, see Fig. 2). All but three men interviewed mentioned hearing Ayahs-messages about the risks of SHS, citing the dangers of polluting their home and damaging the health of their family, particularly their children. Most also remembered the clear direction from the imam within these Ayahs-messages to stop smoking near other people.

*If I smoke, people who are around me are also harmed. Cause when I breathe out the smoke, the people around inhale the oxygen or the air, they are also harmed. They are harmed more than me. Then it is seen, when a child is born or a woman is pregnant, smoking harms her children.*

[Man, SFH intervention, nobody smokes in home at 3-month follow-up]

Noticeably less well recalled by men were Ayahs-messages targeting self-efficacy, coping planning and intention formation (37.5% to 45.0%, see Fig. 2). Just five men who were interviewed mentioned that the imam provided guidance on “how” to change their smoking behaviours, whilst a similar minority declared the imam provided no advice at all.

Finally, the intention was that 100 copies of a short SFH booklet would be distributed in each mosque, thus reaching 3,000 households in total. Imams were unanimous that the booklets were popular, copies were distributed quickly, and more were needed. Some had targeted smokers, elders, or people they considered to be educated who would most benefit.

*We can understand who smokes. We tried to give it to them. Besides them, there are many educated people who want to know about it. We distributed among those educated and smokers.* [Imam 2]

The interview data suggested that reach of the booklet was poor. No men interviewed reported receiving it and some added they could not have read it anyway. Three women mentioned that their sons had brought the booklet home, two of whom could not read.

*We have received it, but we could not understand what the booklet was about, so we have thrown it away. We are women so we don’t understand all these things.*

[Woman, SFH+IAQ, nobody smokes in home at 3-month follow-up]

#### Frequency and reach of IAQ feedback were good. Fidelity was not assessed

Research team records that included a signature from households showed that IAQ feedback was delivered to all 640 households (100%) in that trial arm indicating good intervention frequency. Good reach was also achieved with 98.9% of household leads and 13 of 15 interview participants (men and women) reporting having received the IAQ feedback. Half of interview participants (men and women) mentioned that another family member had received the report. A few commented they could not read the IAQ report, relying on others to do this.

Nobody can read in the home. The youngest daughter read it us twice or three times. After her departure, we were unable get information from it.

[Man, SFH+IAQ, nobody smokes in home at 3-month follow-up]

### Mechanisms of impact

#### SFH intervention acceptability was good. Drivers were new SFH knowledge with corresponding positive attitudes, social norms and intentions. Barriers were a lack of self-efficacy and plans

The male household lead and imam interview data were convergent indicating good acceptability of the SFH intervention. The consensus amongst the men was that listening to the messages in the mosque “*felt good*”, informed them and motivated them to change their smoking behaviours.

*I felt deeply pleased because the message of the imam melts everyone’s heart. I felt like if I could give up smoking from today.*

[Man, SFH intervention, nobody smokes in home at 3-month follow-up]

One exception was a man who was not interested in the intervention, suggesting that he already knew this information anyway.

The imams were also very enthusiastic. Their perception was that the Ayahs-messages were well received by their congregations, and the SFH intervention was useful and appropriate.

*I believe that this is a very useful intervention and it is praiseworthy. The objectives are very helpful for our society and it is a responsibility for us all to ensure that the objectives are properly enforced. From Islamic approach and societal approach, this intervention is praiseworthy on both fronts*. [Imam 4]

They also observed that delivering the messages during Jumu’ah prayer was the right thing to do as that is when the mosque was most crowded, would reach large numbers of people and potentially have greatest impact.

*The Jumu’ah prayer time is the most suitable time for it because what I have seen in my 22 years’ experience as an imam is that approximately 90% of people of our society attends Jumu'ah prayer even though they do not perform the rest of the prayers. The best time to discuss it is the time before Khutbah as there is no chance to discuss these topics after the Jumu’ah prayer. Not all the partakers are present when the Jumu’ah speech starts around 12.25 or 12.30 pm but they are before the Khutbah.* [Imam 5]

The proposed individual drivers of behaviour change were attitudes, self-efficacy, social norms, intention formation and planning (see Fig. 1). Men’s interview accounts clearly illustrated a development in their knowledge and a shift in their attitudes and social norms about SHS, from the messages delivered in the mosque (further confirming the recall data above). In fact, SHS and the risks to others appeared to be new information for most, eliciting beliefs about the social consequences of their smoking, especially the potential harm they were doing to their children. Several participants, both men and women, mentioned having fresh air to breath, healthier children, and no bad smell in the house.

*If I want to keep my children healthy and safe then it is best for me to quit smoking completely. He also said to advise others who smoke to quit as well since it does harm those around you, particularly the children. Smoking is harmful for oneself and their families*. [Man, SFH intervention, still some smoking in home at 3-month follow-up]

*I think that if I quit it will benefit everyone, not just one person. The smoke and smell will not affect anyone if there is no one smoking at all*.

[Man, SFH intervention, still some smoking in home at 3-month follow-up]

Amongst many men, there was evidence of an intention to act, prompted by the words of the imam and a corresponding new awareness of SHS.

*It was mostly due to the hujur’s speech that inspired me. He always speaks to us keeping our best interest in mind. He refers to Hadith so that we know what is best for our Muslim community. I liked his messages very much and realised that it is for the best that I should try to stop smoking at home*.

[Man, SFH+IAQ, lots of smoking in home at 3-month follow-up]

Notably whilst these men appeared motivated to change, they did not speak of ”how” to translate their intention into action or their self-efficacy in doing so. Just one man explicitly spoke of his confidence in creating a SFH, instilled by the imam. Conversely, three men who were not motivated by the imam to change, all alluded to a lack of strategies and low self-efficacy mentioning addiction and stress. One stated that he never listens to the imam because he felt unable to apply this “education” into his life.

*Look everything that the hujur tells is very educative. We all actually know it but how many of us listen to it? If I cannot apply those in my own life, then there is no meaning of this educative lines. I never pay attention to the hujur’s speech*.

[Man, SFH intervention, nobody smokes in home at 3-month follow-up]

#### IAQ feedback acceptability was good. Drivers were new SFH knowledge with corresponding positive attitudes, social norms and intentions. Barriers were a lack of plans

The IAQ machine that measured the air quality in the home, the personalised air quality report and subsequent conversation with the field investigator were well received. They were seen by household leads (men and women) to be educative and prompting intentions to create a SFH.

*I like the way you provide us report. It’s a systematic way. They made us understand very clearly with the help of that report. It was shown how smoking is causing harm. That’s why I liked it most*. [Man, SFH+IAQ, nobody smokes in home at 3-month follow-up]

As with the SFH intervention, interview accounts illustrated a development in SHS knowledge and a shift in beliefs, attitudes and social norms. Approximately half the men and women interviewed spoke of learning that the air pollution was at levels that were dangerous to their family’s health; and the importance of the smoker going outside or away from other people to smoke.

*We learnt from your initiative and nice report. We realized that it actually harms our health or the children’s health. So, it is better not to smoke. Even if I have to smoke, I can do it outside home.* [Man, SFH+IAQ, nobody smokes in home at 3-month follow-up]

This new understanding elicited strong beliefs about the importance of having of a SFH, particularly to improve their children’s health. A few admitted the personalised feedback had “scared” smokers into action.

*After this machine was set here, we felt one kind of fear in us and in our children as well. They are afraid of it thinking, “If we smoke then something bad might happen to us”, so we will not smoke*. [Woman, SFH+IAQ, nobody smokes in home at 3-month follow-up]

All male participants had positive intentions to create a SFH following their IAQ feedback.

*You made me understand the facts while visiting my home and when I saw the facts with proof in my own eyes then I thought it’s better to give up this habit*.

[Man, SFH+IAQ, nobody smokes in home at 3-month follow-up]

Consistent with the SFH intervention, there was no mention of specific strategies that the men planned to use to avoid smoking in the home or negotiation strategies that family members could use.

#### Mixed views on usefulness of SFH intervention. Moderate usefulness of IAQ feedback

Amongst men who reported receiving the SFH intervention 38.2% (SFH) and 79.2% (SFH + IAQ) said it was useful in helping their family achieve a SFH, whilst 60.1% of household leads (men and women) found the IAQ feedback useful.

In describing different levels of smoking in their homes, some interview participants referred to the interventions.

*I used to smoke inside. Now when I buy a cigarette from a tea stall, I smoke beside that place instead. When hujur said this, we heard and forgot. But after getting the machine, I got scared*. [Man, SFH+IAQ, nobody smokes in home at 3-month follow-up]

*Since the machine, I mostly smoke outside, in my shop or where I buy the cigarettes. I plan that in three months my house will be 80% less smoking inside. I still smoke near my children.* [Man, SFH+IAQ, still some smoking in home at 3-month follow-up]

After listening to the hujur’s messages, my son has reduced his smoking in the house. He used to smoke ten times inside and now it’s decreased to three.

[Woman, SFH intervention, still some smoking in home at 3-month follow-up]

Finally, just a small minority of interview participants (men and women) mentioned that they now request other visitors to their home not to smoke indoors.

*I told them that I don’t smoke inside my house, so you are not allowed to smoke here. If you want, you may do this outside of my house*.

[Man, SFH intervention, still some smoking in home at 3-month follow-up]

This had resulted in one woman’s brother no longer coming to the house. However, one man continued to permit “*special guests*” to smoke in his home.

### Context

#### Social context drivers to SFH intervention implementation were in place and important. No context barriers to implementation were reported

The consensus amongst imams was that they had faced no barriers in delivering the SFH intervention. Social context seemed important. Permission from the Islamic Foundation was acknowledged as crucial to demonstrate acceptance of the intervention and a united approach across mosques. Within their own mosques, imams had felt supported by their mosque committees in the form of approval. One valued sharing intervention delivery with a khatib, and another would have liked to have ongoing collaboration about delivery with imams from other mosques.

#### Context barriers/drivers to IAQ feedback implementation were not assessed

IAQ frequency and reach data suggested that there were no context barriers to implementation.

#### Social context drivers to SHS behaviour change were children’s requests. Barriers were a reluctance to request male family members and visitors to smoke outside. (Not) having somewhere to smoke outside was a physical context (barrier) and driver

Social and physical context barriers and drivers to SHS behaviour change emerged predominantly from male household lead interview data. The key social driver to men smoking outside was having children in the home, with children’s direct requests providing further influence.

*It is important when my daughter says, “Father, please do not smoke and even if you need to then smoke outside the home. Do not smoke in front of me.” Is it not an important thing when the daughter calls her father?* [Man, SFH intervention, nobody smokes in home at 3-month follow-up]

Social context barriers were evident. Some women remained reluctant to request male family members to smoke outside seeing this request as “inappropriate”. A few men and women did not want to ask *all* guests to smoke outside. Others were happy to do so, confirming the mixed self-reported behaviour change data above.

*I usually tell them not to smoke inside the house, but if it’s a special guest then they are allowed.*

[Man, SFH intervention, nobody smokes in home at 3-month follow-up]

An additional perspective on social context was offered by several imams. They advocated taking a broader societal approach to enhance message exposure and impact by involving the media and the internet, engaging other institutions such as schools and workplaces, and additional influential community leaders like politicians and celebrities.

*I think that if you can include those who are in charge of making decisions in a society, community leaders, as well as committee of the mosques, then this will be more effective. Political leaders have a lot of influence over many in our society. If you can include them somehow then I think your intervention will have better impact*. [Imam 1]

*If you can look for these celebrities and large gatherings where multiple speakers offer their speech, there are minimum two to three spokesman in these gatherings, you can reach a huge audience by building up relationship with them to briefly include this topic in his speech. He will proceed the discussion according to his rules but if he includes some important facts about smoking, it will be better according to me*. [Imam 5]

Finally, physical context was also a driver and barrier to SHS behaviour change for men. Most readily identified other places they could smoke, mentioning the road, at work or outside the tobacco shop. There were two exceptions. One man complained he had nowhere to smoke outside late at night because the gates to his compound are locked. Another did not want the shame of being seen smoking by other people.

*When I work at night and stay up late, the gates are locked by 11 or 11.30. I don't go out then. I smoke at home*. [Man, SFH+IAQ, still some smoking in home at 3-month follow-up]

*I do not smoke outside at all. If I smoke outside now, people would say, “Uncle, as you are an elderly person, you should not smoke.” It is a matter of shame, thus, I do not smoke at all outside*. [Man, SFH+IAQ, nobody smokes in home at 3-month follow-up]

## Discussion

Our investigation into the implementation, mechanisms of impact and context [25] of the SFH intervention and IAQ feedback uncovered several explanations for their lack of effectiveness in reducing exposure to SHS in the home (when objectively measured). In short, evidence of implementation of the SFH intervention in the mosques was mixed, and good for IAQ feedback. Both interventions had high acceptability but mixed perceptions of usefulness. Household leads described new SFH knowledge with corresponding positive attitudes, social norms and intentions, whilst self-efficacy and plans were lacking. Context for behaviour change was both positive (e.g. children’s requests to smoke outside, places to smoke) and negative (e.g. women’s reluctance to ask men to smoke outside, nowhere for men to smoke outside).

### Strengths and limitations

Our mixed method process evaluation comprised four data sets that were triangulated to elucidate three key process evaluation functions. This approach is recommended as good practice [25, 28], ensured a comprehensive process evaluation, and afforded confidence in our conclusions.

There were some gaps. Context barriers/drivers and fidelity for IAQ delivery were not assessed. The 100% frequency and 98.9% reach data suggest there were limited/no barriers to delivery, and whilst we do not know the quality of the IAQ verbal feedback provided, the IAQ written report was standardised. We have very little interview data from women on their context barriers/drivers to achieving a SFH. Also, our sample of imams interviewed (*n* = 6) and mosques where fidelity assessment was conducted (*n* = 6, 20%) was small. However, they were randomly selected, we captured diversity in their accounts and intervention delivery, and household data were confirmatory. We have no reason to think that other imams' accounts or delivery would be markedly different.

### Why did the interventions not work?

Features of success for both interventions were good acceptability, good frequency of IAQ feedback and moderate to good SFH intervention delivery within Friday Jumu’ah prayers. Moreover, imams reported no context barriers to delivery and important drivers (permission from the Islamic Foundation, support from the mosque) were in place. These positive findings are not unexpected. We engaged stakeholders in our intervention adaptation and development which is accepted good practice [15, 30]. The IAQ feedback was based on a format previously used in Europe [17–22] and adapted for Bangladesh with household lead input. With hindsight we should have considered more carefully how the report would be used by those who cannot read. The SFH intervention was developed using an iterative and collaborative approach (with the Islamic Foundation, imams and household leads) [26] to ensure that it was truly “a religiously inspired approach” [9, p1176] with acceptability and feasibility. Also, key lessons about intervention content (e.g., ensuring that the imams were credible “non-smoking” SHS messengers [7]) and delivery (e.g. support from mosque committees) were gathered from an earlier pilot trial [31]. These informed careful preparation work with mosques and imams to ensure they were ready for intervention delivery, a “success factor” of effective faith-based health promotion programmes [32].

Less positive were findings of poor reach of the SFH intervention and mixed quality of delivery. Only half of household leads recalled receiving the SFH intervention (or their family members receiving it) and no men interviewed had received the booklet. Although Friday prayers are traditionally attended by most Muslim men, the Khutbah sessions delivered before prayers are not mandatory. Anecdotally, attendance may be as low as 10% of the total attendance in Friday prayers which may explain the poor reach. With hindsight, we should probably have been more prescriptive about dissemination to other congregations (including distribution of the SFH booklet), to increase frequency and reach. As an example, a “potentially effective” Korean church-based intervention targeting SHS was more widely embedded across church activities that lasted up to 1.5 h, with dissemination of multiple resources (SHS brochures, quit-smoking guides, SHS stickers, reusable grocery bags, and insulated lunch bags) [33].

Ayahs-messages targeting SHS attitudes and social norms were the self-declared focus of imams, with those targeting attitudes implemented most fully. These were also the Ayahs-messages recalled by male household leads, resulting in new knowledge with a corresponding shift in their SHS attitudes, social norms and intentions to change their SHS behaviours. The SHS health messages e.g. risks to children, were best remembered rather than the corresponding religious text. Even if they had remembered the religious connection, this will only have impacted on motivation [9]. Ayahs-messages that targeted self-efficacy (employing instruction, verbal persuasion and self-talk techniques [34]) and planning (using “if–then” plans [35, 36]) were not remembered and were less well delivered. It seems that imams can confidently educate but lack skills or motivation to deliver strategies to turn knowledge into behaviour. The same outcome was evident for the IAQ feedback, with interview participants self-reporting learning about the risks of SHS at home, changing their attitudes, social norms and being motivated to create a SFH, yet plans for how to do this were absent.

Both interventions were based on well-evidenced behaviour change techniques including those targeting self-efficacy [34] and planning [34–36], yet they were remembered by recipients as educational interventions. It seems likely that men were ill-equipped with confidence, coping and planning skills to overcome significant context barriers and translate positive intentions into behaviour. This hypothesis is consistent with a scoping review of fathers’ experiences of creating a SFH [37] and European evaluation of an SFH intervention [20]. Our interview data with women suggest they found it difficult to request male family members to smoke outside. Other studies reporting women’s inability to negotiate SFHs also report these gendered power interactions [38, 39]. Men-inclusive community interventions (like ours) that aim to change social norms around smoking rather than relying on women to set household boundaries offer potential to improve gender equity as well as health [37, 40]. However, they need to be supported by “gender transformative tobacco control” [41, p796] where gender theory is embedded into public health policy [41]. Overall, it is unsurprising that there was a lack of perceived “usefulness in creating a SFH” for both interventions, and no effect on the SHS exposure in homes (measured by 24-h mean household airborne fine particulate matter (< 2·5 microns in diameter [PM2·5]) concentration) both at 3- and 12-months post-intervention [24].

Literature reviews [42–45] consistently cite promising evidence for faith-based health promotion interventions whilst advocating more rigorous evaluation. Our SFH intervention comprised many “success factors” for effective faith-based programmes [32]. There is also support for IAQ feedback interventions in Europe [16–22]. Our IAQ feedback was an adapted version of these European feedback tools, although our frequency was less than other programmes that incorporate repeat measurement, follow-up visits or phone calls [16–22]. What was different for both interventions is that we did not include one-to-one practical support for behaviour change (including boosting confidence, developing coping and planning skills) which is evident in other faith-based programmes via motivational coaches [33], lay volunteers [32] or faith nurses [42]. We also did not include a motivational interview component [16–22] with the IAQ report. A 2018 review concluded that the effectiveness of educational interventions in reducing SHS exposure was unclear [3]. Whereas combining SHS interventions with smoking cessation support may reduce SHS exposure [18].

Alturki [9] proposes that civil society including Muslim authorities should supplement smoking cessation programmes delivered by health professionals. Unfortunately, in Bangladesh, smoking cessation services are lacking, reflecting poor implementation of the World Health Organization Framework Convention on Tobacco Control (FCTC) [46] Article 14 across LMICs [47]. A further challenge is the weak implementation of SHS legislation (WHO FCTC Article 8) in Bangladesh, again consistent with other LMICs [47, 48]. The WHO [8] and other authors in this field [7, 9] advocate a community-wide strategy where faith-based programmes are ‘one part of a comprehensive overall approach to tobacco control’ [8] including cessation services and good policy. Embedding our two interventions within this wider community approach would seem sensible. One example would be to link with the established network of community health workers who deliver primary care and behaviour change counselling services in Bangladesh, to achieve a “multiplier effect” [49].

## Conclusions

Despite detailed development and adaption work with relevant stakeholders, the SFH intervention and IAQ feedback became educational and motivational but were insufficient to overcome significant context barriers to SHS behaviour change. Future interventions should include practical support for SFH behaviour change. Moreover embedding these into community wide strategies that include practical cessation support and enforcement of SFH legislation is needed.

## Supplementary Information

**Additional file 1. **Linked Ayah-messages and target constructs.

**Additional file 2. **Triangulation matrix for SFH intervention.

**Additional file 3. **Triangulation matrix for IAQ feedback.

## Data Availability

The datasets used and/or analysed during the current study are available from the corresponding author on reasonable request. De-identified individual participant data will be made available from the point of, and up to 5 years after the acceptance for publication of the main findings from the final dataset. These data can be requested from the Principal Investigator (Prof Kamran Siddiqi; kamran.siddiqi@york.ac.uk) and will be shared after the provision of a methodologically sound proposal, and only under a data-sharing agreement that provides for commitment to: using the data only for research purposes and not to identify any individual participant; securing the data using appropriate computer technology; and destroying or returning the data after analyses are completed. The proposals will be assessed and approved by members of the Programme Management Group.

## Abbreviations

FCTC: Framework Convention on Tobacco Control
IAQ: Indoor air quality
LMIC: Low and middle-income countries
MCLASS: Muslim Communities Learning About SHS
SFH: Smoke-free homes
SHS: Second-hand smoke
WHO: World Health Organisation
