# Supplementary material for: “Everything the hujur tells is very educative but if I cannot apply those in my own life then there is no meaning”: a mixed-methods process evaluation of a smoke-free homes intervention in Bangladesh
Source: BMC Public Health. 2022 Oct 11;22:1889. doi: 10.1186/s12889-022-14283-6 (PMC9552417; doi:10.1186/s12889-022-14283-6)
Supplement: Supplementary file 1 — Additional file 1. Linked Ayah-messages and target constructs. [file 12889_2022_14283_MOESM1_ESM.docx]

**Additional file 1.** Linked Ayah-messages and target constructs

| **Cycle** | **Week** | **Ayah** | **Message** | **Construct** |
| --- | --- | --- | --- | --- |
| 1 | 1 | **Surah Al-Maaida - 4 (5:4)**  They ask you, [O Muhammad], what has been made lawful for them. Say, "Lawful for you are [all] good foods.” | Though sometimes people think that smoking helps in some ways, the evidence that smoking, and second-hand smoke cause harm in many ways is clear.  Would Allah permit you something harmful? No! Tobacco is harmful, and hence it is not permissible to Allah. The sin of smoking causes you spiritual as well as physical harm. | Attitude |
| 1 | 2 | **Sura An-Nisaa – 59 (4:59)**  Believers! Obey Allah and obey the Messenger, and those from among you who are invested with authority. | Allah, in his grace, has given us experts who he has been given authority to tell us the facts about what heals us and what harms us.  The evidence from scientists tells us that second-hand smoke contains more than 7,000 chemicals. Hundreds are toxic and about 70 can cause cancer. Second-hand smoke also causes numerous health problems in infants and children.  Will you not listen to the facts? Will you not hear what your Imam says to you? | Attitude |
| 1 | 3 | **Sura Al-Ahzaab – 58 (33:58)**  And those who harm believing men and believing women for [something] other than what they have earned have certainly born upon themselves a slander and manifest sin. | The evidence that second-hand smoke harms other is clear. It can result heart attack, stroke and lung cancer among innocent adults who are exposed to it. And children exposed to second-hand smoke are more prone to have chest infection, sneezing and coughing. Moreover, they have 50% higher chance of having ear infection. Now do you really want to do that to your family members and your children?  Allah also said that – causing harm to others is a manifest sin. | Social norms |

| 1 | 4 | **Sura At-Takaathur – 8 (102:8)**  Then, on that Day, you will be called to account for all the bounties you enjoyed. | These messages to you are part of Allah’s bounty to you. But you need to make a commitment to enjoy his bounty. This means committing to either quitting or smoking outside. If you are going to do this, you need to make a plan.  For planning to stop smoking at home, commit that if you reach for a cigarette – then leave the house before you light it. And for planning to quit smoking completely, commit that if you feel like smoking, then pray 2 rakat salat instantly. | Intention formation  (and prompt action planning) |
| --- | --- | --- | --- | --- |
| 1 | 5 | **Sura Ar-Ra’d – 11 (13:11)**  The fact is that Allah does not change a people's lot unless they themselves change their own characteristics | You can trust Allah to help you, but to receive that support, you must take a step by yourself in faith. Trust that Allah will give you everything you need.  You can find it difficult to stop smoking at home. But if YOU cannot make this simple change of behaviour for the sake of your family members, how can you expect Allah will help them in other ways? So, you need to make your plan of smoking outside home. For example, if you feel like smoking when you are at home – then leave the house before you light it. You can plan to remove your last cigarette before you come home. | Self-efficacy  (prompt Action Planning) |
| 1 | 6 | **Surah Al-Maaida - 9 (5:9)**  Allah has promised those who believe and do righteous deeds [that] for them there is forgiveness and great reward. | Allah knows you, Allah knows everything. He knows that you will need his forgiveness. Be quick to come to him. Trust that he will be with you as you come back to the right path.  So make a plan that if you lapse, then you will call on Allah for forgiveness and recommit yourself and rehearse your plans. | Coping planning |

| 2 | 7 | **Sura Al Maaida – 90 (5:90)**  Believers! Intoxicants, games of chance, idolatrous sacrifices at altars, and divining arrows are all abominations, the handiwork of Satan. So turn wholly away from it that you may attain to true success. | Tobacco is toxic. Your body becomes reliant on nicotine. It doesn’t relieve stress. It only relieves withdrawal syndrome from your addiction.  Tobacco is the handiwork of Satan. Do you want true success? Turn away wholly from tobacco. | Attitude |
| --- | --- | --- | --- | --- |
| 2 | 8 | Surah Al-Maaida - 100 (5:100)  Say, "Not equal are the evil and the good, although the abundance of evil might impress you." So, fear Allah, O you of understanding, that you may be successful. | Some of you may believe that smoking is good because it helps keep you warm, or stops you getting fat, or manage your stress. But Allah, in his grace, has given us eye to see, ears to hear and a mind to enquire. What do the experts tell us? Experts tell us that it does nothing but harm you and those who are staying beside you when you smoke. The only relief you feel getting after smoking is the relief from withdrawal syndrome which we mistakenly think as stress relief. | Attitude |
| 2 | 9 | **Sura At-Baqara – 195 (2:195)**  And do good; indeed, Allah loves the doers of good. | Globally 600 thousand people die every year due to exposure to second-hand smoke. Those who smoke around us are directly causing harm to us though they are often not aware of the harm they are causing. Hence, we need to be aware and careful about smoking inside home and in front of others.  We need to talk to others about the harm of smoking and second-hand smoke. We need to save our families from this harm. Allah also loves those who does good things. | Social norms |
| 2 | 10 | **Surah Ash-Shams – 7-10 (91:7-10)**  And [by] the soul and He who proportioned it. And inspired it [with discernment of] its wickedness and its righteousness, He has succeeded who purifies it, and he has failed who instils it [with corruption]. | Allah has given you wisdom, but to remember it, you have to act on it. Only then you and others will be benefitted by that.  If you are going to do something, you need to make a plan. For example, if you reach for a cigarette when you are at home – then leave the house before you light it. And for quitting smoking, you should plan like this - if you feel the urge to do smoke, pray 2 rakat salat instantly. | Intention formation |

| 2 | 11 | **Surah At-Taghaabun - 16 (64:16)**  So, fear Allah as much as you are able and listen and obey and spend [in the way of Allah]; it is better for yourselves. And whoever is protected from the stinginess of his soul - it is those who will be the successful. | Those who smoke can find it difficult to quit smoking or they can find it hard to go outside home every time they want to smoke. But believe it, Allah will help you if you wish to listen to him. One can make simple plans to overcome such issues. Just commit to yourself and others (if you can) that whenever you feel the urge of smoking, go outside home to light it or pray 2 rakat salat instantly. | Self-efficacy  (prompt Action Planning) |
| --- | --- | --- | --- | --- |
| 2 | 12 | **Surah Al-Hajj - 77 (22:77)**  Oh you who have believed, bow and prostrate and worship your Lord and do good - that you may succeed | Allah knows best about his creatures. He understands that we may do things that will harm us and others. That is why, he encouraged us to enjoy all that is good and forbid all that is evil and keep patience in times of affliction.  We must remind ourselves these words of Allah again and again. We must try to make our habits safe for others. We must remember the possible harms of our behaviour to others like smoking at home and repetitively plan to keep us and our families safe from its harm. | Coping planning |
