# Supplementary material for: “Everything the hujur tells is very educative but if I cannot apply those in my own life then there is no meaning”: a mixed-methods process evaluation of a smoke-free homes intervention in Bangladesh
Source: BMC Public Health. 2022 Oct 11;22:1889. doi: 10.1186/s12889-022-14283-6 (PMC9552417; doi:10.1186/s12889-022-14283-6)
Supplement: Supplementary file 2 — Additional file 2. Triangulation matrix for SFH intervention. [file 12889_2022_14283_MOESM2_ESM.docx]

**Additional File 2:** Triangulation matrix for SFH intervention

| **Meta-theme** | | | **Household lead interviews**  **(N=20 men, N=10 women)** | **Imam interviews (N=6)** | **Household lead questionnaire (N=848 men, N=52 women)** | **Fidelity**  **(N=6 mosques)** | **Research team records** | **Level of congruence** | **Conclusion** |
| --- | --- | --- | --- | --- | --- | --- | --- | --- | --- |
|  | | **SFH intervention** | | | | | | |  |
| Implementation | Frequency | |  | 4/6 (66.7%) reported delivering all 12 weeks. All reported distributing the SFH booklet. |  |  | 29/30 (96.7%) mosques reported delivering all 12 weeks. | Complementary | Moderate to good frequency of intervention |
|  | Fidelity | |  | All had delivered the intervention during Friday Juma’ah prayers (as per guidance). Consensus that had shared “most of the Ayahs-messages”.  Most focused on telling congregation about risks (Ayahs-messages targeting attitudes and social norms). |  | Mean fidelity score 19.6 (SD 2.51, range 16-22 of maximum 24).  Ayahs-messages-linked to attitudes 75.0% fully implemented, self-efficacy/action planning and coping planning 66.7% fully implemented, social norms and intention formation/action planning 50.0% fully implemented. |  | Complementary | Mixed levels of fidelity. Ayahs-messages targeting attitudes were most often fully implemented. |

|  | Reach | Majority of men recalled hearing Ayahs-messages during Friday Jumu’ah prayers. Majority of women reported that their partners/sons had received the same.  Men recalled Ayahs-messages on risks of SHS (attitudes, social norms). Less well recalled were Ayahs-messages targeting self-efficacy, coping planning, and intention formation.  No men had received the SFH booklet. 3 women had sons who received the booklet but 2 could not read it. |  | 49.4% (SFH) and 55.5% (SFH+IAQ) of men had received the SFH intervention. Of these, 99.5% (SFH) and 99.6% (SFH+IAQ) received this during Friday Jumu’ah prayers.  SFH 51.5% (SFH) and 52.6% (SFH+IAQ) of women reported family members receiving the SFH intervention, during Friday Jumu’ah prayers (100%)  80%+ of these men recalled Ayahs-messages targeting attitudes and social norms. Just 37.5-45% recalled those targeting self-efficacy, coping planning, and intention formation. |  |  | Complementary | Poor intervention reach. For those who it did reach, Ayahs-messages targeting attitudes and social norms had the best reach. |
| --- | --- | --- | --- | --- | --- | --- | --- | --- |

| Mechanisms of impact | Acceptability of the intervention | Majority view amongst men that SFH messages were informative and motivational. | Consensus that SFH intervention acceptable, appropriate and well received by congregation. |  |  |  | Convergent | Good acceptability of intervention |
| --- | --- | --- | --- | --- | --- | --- | --- | --- |
|  | Barriers and drivers to change (related to the individual) | Majority view amongst men that knowledge about SHS was new and changed their SHS attitudes, social norms and intended to smoke outside. They did not mention plans. Minority that was not motivated attributed this to lack of self-efficacy. |  |  |  |  | N/A | Drivers to change were new SFH knowledge with corresponding positive attitudes, social norms and intentions.  Barriers were a lack of self-efficacy and plans. |
|  | Usefulness of the intervention^a^ | In describing different levels of smoking in their homes, some interview participants referred to the intervention. |  | 38.2% (SFH alone) and 79.2% (SFH+IAQ) of men reported that the SFH intervention was useful in achieving a SFH |  |  | Convergent | Mixed views on usefulness of SFH intervention. |

| Context | Impact on SFH implementation |  | Consensus that felt supported by mosque committee. Permission from Islamic Foundation seen as important. No barriers to delivery. |  |  |  | N/A | Social context drivers were in place and important for implementation.  No context barriers reported. |
| --- | --- | --- | --- | --- | --- | --- | --- | --- |
|  | Impact on SHS behaviour change^b^ | Minority of men/women spoke of children motivating men to smoke outside. Conversely social norms prevented some asking visitors to smoke outside and women to request this of male family members.  Majority of men could identify other places to smoke. A minority could not. | Some imams advocated a wider societal approach to achieve behaviour change. |  |  |  | Complementary | Social and physical context barriers and drivers to creating a SFH were evident. |

*Note.* Convergent = in agreement, complementary = partial agreement, contradictory = disagreement, silent = findings do not occur in a dataset but may have been expected to do so [29]. ^a^Same usefulness of the intervention data (mechanisms of impact) and ^b^impact on outcomes data (context) are reported in Additional Files 2 and 3.
