# Supplementary material for: “Everything the hujur tells is very educative but if I cannot apply those in my own life then there is no meaning”: a mixed-methods process evaluation of a smoke-free homes intervention in Bangladesh
Source: BMC Public Health. 2022 Oct 11;22:1889. doi: 10.1186/s12889-022-14283-6 (PMC9552417; doi:10.1186/s12889-022-14283-6)
Supplement: Supplementary file 3 — Additional file 3. Triangulation matrix for IAQ feedback. [file 12889_2022_14283_MOESM3_ESM.docx]

**Additional File 3:** Triangulation matrix for IAQ feedback

| **Meta-theme** | | **Household lead interviews**  **(N=20 men, N=10 women)** | **Household lead questionnaire (N=848 men, N=52 women)** | **Research team records** | **Level of congruence** | **Conclusion** |
| --- | --- | --- | --- | --- | --- | --- |
| Implementation | Frequency |  |  | IAQ feedback delivered to all 640 households | N/A | Good frequency of intervention |
|  | Fidelity |  |  |  | No data | No data |
|  | Reach | Majority remembered receiving the IAQ feedback, half reported another household member receiving the report. Minority commented that they could not read it. | 98.9% of households received the IAQ feedback |  | Convergent | Good reach of intervention |
| Mechanisms of impact | Acceptability of the intervention | Consensus that IAQ feedback was informative and motivational |  |  |  | Good acceptability of intervention |
|  | Barriers and drivers to change (related to the individual) | Consensus that that knowledge about SHS was new and changed their SHS attitudes, social norms and intended to create a SFH.  No one mentioned planning for this. |  |  |  | Drivers to change were new SFH knowledge with corresponding positive attitudes, social norms and intentions.  Barriers were a lack of plans. |
|  | Usefulness of the intervention^a^ | In describing different levels of smoking in their homes, some interview participants referred to the intervention. | 60.1% of households reported that the IAQ feedback was useful in achieving a SFH |  | Convergent | Moderate usefulness of IAQ intervention. |
| Context | Impact on implementation |  |  |  |  | No data |
|  | Impact on SHS behaviour change^b^ | Minority of men/women spoke of children motivating men to smoke outside, conversely social norms prevented some asking visitors to smoke outside.  Majority of men could identify other places to smoke. A minority could not. |  |  | N/A | Social and physical context barriers and drivers to creating a SFH were evident. |

*Note.* Convergent = in agreement, complementary = partial agreement, contradictory = disagreement, silent = findings do not occur in a dataset but may have been expected to do so [29]. ^a^Same usefulness of the intervention data (mechanisms of impact) and ^b^impact on outcomes (context) data are reported in Additional Files 2 and 3.
